# Intuitive eating has reduced the chances of being overweight in university students during the COVID-19 pandemic

**DOI:** 10.3389/fnut.2024.1329788

**Published:** 2024-02-15

**Authors:** Caroline Rodrigues Souto, Luciane Bresciani Salaroli, Gabriela Grillo da Silva, Monica Cattafesta, Yasmin Franco Rodrigues Silva, Fabíola Lacerda Pires Soares

**Affiliations:** ^1^Graduate Program in Nutrition and Health, Federal University of Espírito Santo, Vitória, Brazil; ^2^Graduate Program in Public Health, Federal University of Espírito Santo, Vitória, Brazil

**Keywords:** COVID-19, student health, eating behavior, nutritional status, intuitive eating

## Abstract

**Introduction:**

University students have been particularly affected during the COVID-19 pandemic, and several sociodemographic and behavioral factors may be associated with the risk of overweight in this population. The aim of this study was to assess the impact of the pandemic on the eating behavior and nutritional status of university students, and the factors associated with these changes, especially the role of intuitive eating in this process.

**Methods:**

This is a repeated measures observational study with data collected in the first and third year of the COVID-19 pandemic in Brazil, from students over the age of 18 in the undergraduate programs of a federal university in southeastern Brazil. The survey was conducted using an online form. Eating behavior was assessed using the “Intuitive Eating Scale–2”.

**Results:**

251 university students took part, most of them female with a median age of 22. There was an increase in body mass index (BMI) and intuitive eating score between the two periods. In the first year of the pandemic, being female and using tobacco reduced the chances of students being overweight. Living in a marital relationship, worrying about weight gain and body dissatisfaction increased the chances of this outcome. In the third year of the pandemic, it was observed that practicing restrictive diets, having inadequate body perception, worrying about weight gain and living in a marital relationship increased the chances of students being overweight. Being female and eating more intuitively, in line with bodily needs, reduced these odds, demonstrating a protective role in this scenario.

**Conclusion:**

There was an increase in BMI and intuitive eating score during the pandemic. Sociodemographic, lifestyle and behavioral variables had both a positive and negative influence on nutritional status. Intuitive eating was shown to be a protective factor during this period, reducing the chances of being overweight in this population. Thus, more intuitive eating may favor greater weight stability, and may, therefore, have helped to reduce the impact of the pandemic on weight gain. In this way, people who ate more intuitively partially resisted the context that favored weight gain (stress, changes in diet and physical inactivity).

## 1 Introduction

University students entering academic life are at a time of transition in their lives, which makes them prone to emotional imbalances. Entering university generates challenging experiences due to major changes, and symptoms of stress and depression can be commonly present in this process (1). Changes in eating behavior generated by stress most often drive individuals to eat more (2), and among the various stressors that can alter eating behavior, the COVID-19 pandemic has recently stood out, with a major impact on the entire world population.

The COVID-19 pandemic was declared a public health emergency of national importance in February 2020 (3). Given the increase in the number of cases, in March of that same year the actions implemented in other countries began to be practiced in Brazil as well, with the aim of substantially reducing the spread of the disease (4). Non-essential economic activities were paralyzed, including the implementation of exceptional measures in higher education institutions (5).

The effect of the measures taken and restrictions imposed had repercussions on various aspects, including psychological, emotional, economic and physical (6). With regard to physical aspects, the level of physical activity has been considerably reduced by the majority of the population (7). In addition, the emotional and psychological changes caused by the general panorama of the pandemic (anxiety, sadness, loneliness) have also contributed to excess calorie consumption, and consequently weight gain (8–10).

In a study carried out with university students in another setting, it was found that students with higher levels of stress had higher scores for emotional eating and uncontrolled eating behavior, and a higher frequency of consumption of snacks (1). Individuals with uncontrolled eating behavior tend to consume not only high-calorie, hyper-palatable foods, but also excessive amounts of them, ingesting more energy than their bodies need (11), which consequently leads to weight gain (8).

In contrast to these negative changes in eating behavior, intuitive eating stands out, which is characterized by food consumption based on internal signals of hunger and satiety (12). It is a pattern of eating behavior based on the body’s ability to regulate its own nutritional needs (13). Its concept involves four dimensions (sub-scales): Unconditional Permission To Eat (UPE); Eating for Physical Rather than Emotional Reasons (EPR); Reliance on Hunger and Satiety Cues (RHSC); and Body-food Choice Congruence (B-FCC) (12).

More intuitive eating has a low relationship with preoccupation with food and a strong physical relationship with the body (13). Camilleri et al. (14) showed that more intuitive eating, especially based on physical rather than emotional reasons, trust in hunger and satiety signals and unconditional permission to eat, was associated with a lower body mass index (BMI) in men and women. In addition, other studies have associated intuitive eating with a positive body image and a reduction in depressive symptoms (15, 16). Studies have also shown a negative correlation between intuitive eating and BMI in young adults, adolescents and university students (17, 18).

Considering, therefore, the various changes caused by the COVID-19 pandemic in the physical and mental health status of the population, especially university students, and the scarcity of national studies evaluating this panorama, the objective of this study was to evaluate the impact of the pandemic on the eating behavior and nutritional status of students at a federal university in southeastern Brazil, and the factors associated with these changes, especially the role of intuitive eating in this process. The hypothesis of this study is that the COVID-19 pandemic has acted as an important factor in social, physical and psychological changes, with unfavorable effects on eating behavior and consequently on the nutritional status of university students, and that a more intuitive diet may have had a protective effect against negative changes in this situation.

## 2 Materials and methods

### 2.1 Participants and procedures

This was a repeated measures observational study. The population consisted of 251 students from a university in southeastern Brazil, aged over 18 and of both sexes. Data was collected using semi-structured questionnaires applied online (self-reported data). Recruitment took place via institutional e-mail and dissemination on social networks. Inclusion criteria included students regularly enrolled in any face-to-face undergraduate course, over the age of 18, and who agreed to the Free and Informed Consent Form (FICF). Pregnant and breastfeeding women were excluded from the study due to the physiological changes common to this group, which could directly interfere with eating behavior and body weight.

The first stage of the research covered the initial period of social distancing measures in Brazil and the suspension of face-to-face activities at UFES, with collection carried out in the first year of the pandemic (between May and June 2020). The second collection took place in March 2022 (the third year of the pandemic), which included the end of social distancing measures and the return of face-to-face activities at the university.

### 2.2 Measures

Sociodemographic data was collected (sex, age, marital status, race/color, family income and education of the head of the family); academic data (course enrolled in); anthropometric data (weight and height); lifestyle data (alcohol intake, tobacco use and physical activity); medical history data (practice of restrictive diets and concern about body weight) and behavioral data, including assessment of negative changes in mood (anxiety, sadness, anger, etc.), body perception and satisfaction and intuitive eating. Anthropometric data was obtained based on the current weight (kg) and height (cm) reported. BMI was calculated from this data and classified according to the criteria of the World Health Organization (19). The students were instructed to consider the period up to thirty days back from the date they filled in the form.

Body perception and satisfaction were assessed using the Silhouette Scale by Kakeshita et al. (20), validated for digital use by Freire and Fisberg (21), which consists of a scale of silhouettes developed for assessment in Brazilian children and adults of both sexes. Participants were instructed to look at the digital scale and answer the following questions: “Point to the figure that best represents your current body, which in this study was called Current BMI (BMI-C)”; then “Point to the figure that best represents the body you would like to have, which was called Desired BMI (BMI-D)” (21). The Real BMI (BMI-R) was defined as weight divided by height squared, according to the data provided by the participant. The perception and satisfaction / dissatisfaction with body image was assessed according to the discrepancies between the selected figures. If the difference between the figures was not equal to zero, the individual was classified as having “inadequate body perception” (BMI-C versus BMI-R—when assessing perception) and dissatisfied with body image (BMI-C versus BMI-D—when assessing satisfaction/dissatisfaction) (21).

Eating behavior was assessed using the Intuitive Eating Scale—2 (IES-2), developed by Tylka and Kroon Van Diest (12) and translated into Portuguese by da Silva et al. (22). This scale consists of questions about eating attitudes involving intuitive eating, formulated with 23 items in order to assess an individual’s tendency to follow their physiological hunger and satiety signals (23). The items are classified on a 5-point scale, ranging from 1 (never) to 5 (always), with seven items coded inversely. The analysis of each subscale (UPE, EPR, RHSC and B-FCC) was based on the sum of the item scores, divided by the number of items in each subscale, leading to a possible range of 1 to 5. The evaluation was also carried out by calculating the total score (sum of the scores for all the items divided by 23).

### 2.3 Statistical analysis

Means (with standard deviation) or medians (with interquartile range) and absolute and relative frequencies were used to describe the study variables. The chi-square test or Fisher’s exact test (categorical variables) were used to test the association between the dependent variable and the independent variables at each stage. Fisher’s exact test was chosen when the expected values in the table cells were less than five or when the sum of the column values was less than twenty. The Student’s t-test or Mann–Whitney test was also used to analyze numerical variables. To compare the two collection stages, the difference between the proportions was analyzed using McNemar’s test, the difference between the means using the paired T-test and between the medians using the Wilcoxon test. The significance level for all tests was 5%.

To quantify the participation of the independent variables in the outcome (nutritional status) at each of the collection stages, binary logistic regression was carried out, including in the model the independent variables with significance of less than 5% (*p* ≤ 0.05) in the bivariate association tests. The assumptions of absence of multicollinearity, minimum number for analysis (10 to 20 per independent variable and five cases in each variable category) and absence of outliers were taken into account. We also considered the fit of the model according to the Hosmer-Lemeshow test. The model adjustment was performed only among the variables included in the analysis (i.e., those with *p* ≤ 0.05 in the bivariate tests). Odds ratios and their respective confidence intervals were estimated for each stage.

### 2.4 Ethical aspects

The research was approved by the Research Ethics Committee of the Federal University of Espirito Santo (UFES) through Opinion No. 4.022.658, dated May 12, 2020, and No. 4.080.199, dated June 9, 2020. Participation was voluntary and consent was given when participants ticked “I accept” on the FICF.

## 3 Results

A total of 251 students took part in the study, with a median age of 22 years, and a predominance of females (*n* = 182; 72.5%). Most were not in a marital relationship (*n* = 222; 88.4%), did not drink alcohol (*n* = 129; 51.4%) and did not use tobacco (*n* = 239; 95.2%). The majority of the sample were students enrolled in non-health courses (*n* = 158; 62.9%), of white race/color (*n* = 123; 50.4%) and whose head of household had up to high school education (*n* = 101; 40.2%). A greater number of students had a family income of more than two minimum wages (*n* = 119; 50.6%) (Table 1).

**Table 1:** Sociodemographic, academic and lifestyle data of undergraduate students at a university in southeastern Brazil in the first year of the COVID-19 pandemic, Vitória, 2020.

Variables	*N*	%
**Age—median (minimum–maximum)**	22 (17–68)	–
**Sex**		
Male	69	27.5
Female	182	72.5
**Marital status**		
Do not live maritally	222	88.4
Live maritally	29	11.6
**Alcohol use**		
No	129	51.4
Yes	122	48.6
**Tobacco use**		
No	239	95.2
Yes	12	4.8
**Graduation area**		
Health	93	37.1
No health	158	62.9
**Race/skin color**^**1**^		
White	123	50.4
Black	31	12.7
Brown	87	35.7
Oriental/Indigenous	3	1.2
**Education of the head of the family**		
Did not study/Primary complete	64	25.5
Secondary complete	101	40.2
Complete higher education/Post-graduate	86	34.3
**Family income (minimum wage)**^**2**^		
≤ 2 minimum wage	116	49.4
> 2 minimum wage	119	50.6

*N* = 251. ^1^*N* = 244. ^2^*N* = 235. Minimum wage = R$1045.00.

In the comparative analysis between the data obtained in the first and third year of the pandemic (Table 2), the results show an increase in body weight and BMI (*p* = 0.002 and *p* = 0.003, respectively), without significant alteration in overall classification of the studied population nutritional status. The data also show that the proportion of individuals who started cooking more in the first year of the pandemic decreased when this data was evaluated in the second collection (*p* = 0.002), while the proportion of those who started ordering more food by delivery increased significantly (*p* = 0.001). There was also a significant reduction in the proportion of individuals who reported negative changes in mood (*p* < 0.001) and in the practice of restrictive diets (*p* < 0.001). With regard to intuitive eating, both the total score (*p* = 0.002) and the UPE (*p* = 0.024) and B-FCC (*p* = 0.001) subscales increased significantly between the two periods.

**Table 2:** Physical activity, nutritional and behavioral data of undergraduate students at a university in southeastern Brazil in the first and third year of the COVID-19 pandemic, Vitória, 2020–2022.

Variables	First year	Third year	*p*-value
	*N* (%)	*N* (%)	
**Physical activity**			0.086
No	107 (42.6)	92 (36.7)	
Yes	144 (57.4)	159 (63.3)	
Body weight*^a^	64 ± 23	66 ± 25	**0.002**
BMI*^a^	23.01 ± 6.68	23.57 ± 7.50	**0.003**
**Nutritional status**			0.099
Underweight/Eutrophic	157 (62.5)	147 (58.6)	
Overweight/Obese	94 (37.5)	104 (41.4)	
**Started cooking more**			**0.002**
No	62 (24.7)	92 (36.7)	
Yes	189 (75.3)	159 (63.3)	
**Ordered more food by delivery**			**0.001**
No	179 (71.3)	145 (57.8)	
Yes	72 (28.7)	106 (42.2)	
**Negative mood changes**			**<0.001**
No	52 (20.7)	85 (33.9)	
Yes	199 (79.3)	166 (66.1)	
**Practice of restrictive diets**			**<0.001**
No	155 (61.8)	196 (78.1)	
Yes	96 (38.2)	55 (21.9)	
**Concern about weight gain**			0.374
No	94 (37.5)	102 (40.6)	
Yes	157 (62.5)	149 (59.4)	
**Body perception**			0.341
Adequate	42 (16.7)	31 (12.4)	
Inadequate	209 (83.3)	220 (87.6)	
**Body dissatisfaction**			0.280
No	31 (12.4)	39 (15.5)	
Yes	220 (87.6)	212 (84.5)	
**Intuitive eating – Total score**^**b**^	3.35 ± 0.58	3.45 ± 0.64	**0.002**
Intuitive eating – UPE Subscale*^a^	3.67 ± 0.84	3.83 ± 0.84	**0.024**
Intuitive eating – EPR Subscale*^a^	3.13 ± 1.38	3.38 ± 1.37	0.223
Intuitive eating – RHSC Subscale*^a^	3.33 ± 1.16	3.33 ± 1.37	0.173
Intuitive eating – B-FCC Subscale*^a^	3.33 ± 1.00	3.67 ± 1.00	**0.001**

McNemar test. ^a^Wilcoxon test. ^b^Paired *t*-test. *Data expressed as p50 ± interquartile range. In bold: statistically significant difference (*p* < 0.05). BMI: body weight index. B-FCC: body-food choice congruence. EPR: eating for physical rather than emotional reasons. RHSC: reliance on hunger and satiety cues. UPE: unconditional permission to eat.

In the first year of the pandemic (Table 3) were associated with nutritional status: sex (*p* = 0.017), marital status (*p* < 0.001), undergraduate major (*p* = 0.017), tobacco use (*p* = 0.035), the habit of cooking more (*p* = 0.008), the practice of restrictive diets (*p* < 0.001), concern about weight gain (*p* < 0.001), body perception (*p* = 0.010) and body dissatisfaction (*p* < 0.001). In addition, both the total score (*p* = 0.013) and the UPE subscale score (*p* = 0.010) on the intuitive eating scale were lower in individuals who were overweight or obese, indicating less intuitive eating in this group.

**Table 3:** Sociodemographic, academic, lifestyle and behavioral data distributed according to nutritional status of undergraduate students at a university in southeastern Brazil in the first year of the COVID-19 pandemic, Vitória, 2020.

Variables	Nutritional status (first year)			
	Underweight/Eutrophic	Overweight/Obese	*p*-value	Total
	*N* (%)	*N* (%)		*N* (%)
**Sex**			**0.017**	
Male	35 (22.3)	34 (36.2)		69 (27.5)
Female	122 (77.7)	60 (63.8)		182 (72.5)
**Marital status**			**<0.001**	
Do not live maritally	152 (98.7)	70 (74.5)		222 (88.4)
Live maritally	5 (1.3)	24 (25.5)		29 (11.6)
**Race/skin color**^**1**^^a^			0.515	
White	72 (48)	51 (54.2)		123 (50.4)
Black	21 (14)	10 (10.6)		31 (12.7)
Brown	56 (37.3)	31 (33)		87 (35.6)
Oriental/Indigenous	1 (0.7)	2 (2.2)		3 (1.3)
**Education of the head of the family**			0.762	
Did not study/Primary complete	44 (28)	20 (21.3)		64 (25.5)
Secondary complete	62 (39.5)	39 (41.5)		101 (40.2)
Complete higher education/Post-graduate	51 (32.5)	35 (37.2)		86 (34.3)
**Family income (minimum wage)**^**2**^			0.149	
≤ 2 minimum wage	74 (51.4)	42 (46.1)		116 (49.4)
> 2 minimum wage	70 (48.6)	49 (53.9)		119 (50.6)
**Graduation area**			**0.017**	
Health	67 (42.7)	26 (27.6)		93 (37)
No health	90 (57.3)	68 (72.4)		158 (63)
**Alcohol use**			0.388	
No	84 (53.5)	45 (47.9)		129 (51.4)
Yes	73 (46.5)	49 (52.1)		122 (48.6)
**Tobacco use**^a^			**0.035**	
No	146 (93)	93 (98.9)		239 (95.2)
Yes	11 (7)	1 (1.1)		12 (4.8)
**Physical activity**			0.585	
No	69 (44)	38 (40.4)		107 (42.6)
Yes	88 (66)	56 (59.6)		144 (57.4)
**Started cooking more**			**0.008**	
No	30 (19.1)	32 (34)		62 (24.7)
Yes	127 (80.9)	62 (66)		189 (75.3)
**Ordered more food by delivery**			0.765	
No	113 (72)	66 (70.2)		179 (71.3)
Yes	44 (28)	28 (29.8)		72 (28.7)
**Negative mood changes**			0.257	
No	29 (18.5)	23 (24.5)		52 (20.7)
Yes	128 (81.5)	71 (75.7)		199 (79.3)
**Practice of restrictive diets**			**<0.001**	
No	111 (70.7)	44 (46.8)		155 (61.7)
Yes	46 (29.3)	50 (53.2)		96 (38.3)
**Concern about weight gain**			**<0.001**	
No	80 (51)	14 (14.9)		94 (37.5)
Yes	77 (49)	80 (85.1)		157 (62.5)
**Body perception**			**0.010**	
Adequate	28 (17.8)	6 (6.4)		34 (13.5)
Inadequate	129 (82.2)	88 (93.6)		217 (86.5)
**Body dissatisfaction**			**<0.001**	
No	28 (17.8)	3 (3.2)		31 (12.4)
Yes	129 (82.2)	91 (96.8)		220 (87.6)
**Intuitive eating – total score**^**b**^	3.42 ± 0.59	3.23 ± 0.55	**0.013**	
**Intuitive eating – UPE subscale** ^c^*	3.83 ± 0.84	3.58 ± 0.83	**0.010**	
**Intuitive eating – EPR subscale** ^c^*	3.25 ± 1.37	3.06 ± 1.37	0.137	
**Intuitive eating – RHSC subscale**^c^*	3.33 ± 1.16	3.25 ± 1.08	0.380	
**Intuitive eating – B-FCC subscale** ^c^*	3.67 ± 1.16	3.33 ± 1.33	0.072	

Chi-square test. ^a^Fisher’s exact test. ^b^Student *t*-test. ^c^Mann-Whitney test. *Data expressed as p50 ± interquartile range. In bold: statistically significant difference (*p* < 0.05). *N* = 251. ^1^*N* = 244. ^2^*N* = 235. B-FCC: body-food choice congruence. EPR: eating for physical rather than emotional reasons. RHSC: reliance on hunger and satiety cues. UPE: unconditional permission to eat.

In the third year of the pandemic (Table 4), sex (*p* = 0.016), marital status (*p* < 0.001), alcohol intake (*p* = 0.015), practice of restrictive diets, concern about weight gain, perception and body dissatisfaction (all with *p* < 0.001) were associated with nutritional status. Regarding intuitive eating, the scores on the EPR, RHSC and B-FCC subscales were significantly lower (*p* < 0.001) in overweight or obese individuals, indicating that they eat less intuitively.

**Table 4:** Sociodemographic, academic, lifestyle and behavioral data distributed according to nutritional status of undergraduate students at a university in southeastern Brazil in the third year of the COVID-19 pandemic, Vitória, 2022.

Variables	Nutritional status (third year)			
	Underweight/Eutrophic	Overweight/Obese	*p*-value	Total
	*N* (%)	*N* (%)		*N* (%)
**Sex**			**0.016**	
Male	32 (21.8)	37 (35.6)		69 (27.5)
Female	115 (78.2)	67 (64.6)		182 (72.5)
**Marital status**			**<0.001**	
Do not live maritally	141 (95.9)	81 (77.9)		222 (88.4)
Live maritally	6 (4.1)	23 (22.1)		29 (11.6)
**Race/skin color**^**1**,^^a^			0.614	
White	67 (47.5)	56 (54.4)		123 (50.4)
Black	17 (12)	14 (13.6)		31 (12.7)
Brown	55 (39)	32 (31)		87 (35.6)
Oriental/Indigenous	2 (1.4)	1 (1.0)		3 (1.3)
**Education of the head of the family**			0.491	
Did not study/Primary complete	40 (27.2)	24 (23.1)		64 (25.5)
Secondary complete	58 (39.5)	43 (41.3)		101 (40.2)
Complete higher education/Post-graduate	49 (33.3)	37 (35.6)		86 (34.3)
**Family income (minimum wage)**^**2**^			0.241	
≤ 2 minimum wage	69 (50.7)	47 (47.5)		116 (49.4)
> 2 minimum wage	67 (49.3)	52 (52.5)		119 (50.6)
**Graduation area**			0.142	
Health	60 (40.8)	33 (31.7)		93 (37)
No health	87 (59.2)	71 (68.3)		158 (63)
**Alcohol use**			**0.015**	
No	85 (57.8)	44 (42.3)		129 (51.4)
Yes	62 (42.2)	60 (57.7)		122 (48.6)
**Tobacco use**^a^			0.766	
No	139 (94.5)	100 (96.1)		239 (95.2)
Yes	8 (5.5)	4 (3.9)		12 (4.8)
**Physical activity**			0.407	
No	57 (38.8)	35 (33.6)		92 (36.6)
Yes	90 (61.2)	69 (66.4)		159 (63.4)
**Started cooking more**			0.444	
No	51 (34.7)	41 (39.4)		92 (36.6)
Yes	96 (65.3)	63 (60.6)		159 (63.4)
**Ordered more food by delivery**			0.984	
No	85 (57.8)	60 (57.7)		145 (57.7)
Yes	62 (42.2)	44 (42.3)		106 (42.3)
**Negative mood changes**			0.833	
No	49 (33.3)	36 (34.6)		85 (33.9)
Yes	98 (66.7)	68 (65.4)		166 (66.1)
**Practice of restrictive diets**			**<0.001**	
No	127 (86.4)	69 (66.3)		196 (78.1)
Yes	20 (13.6)	35 (33.7)		55 (21.9)
**Concern about weight gain**			**<0.001**	
No	82 (55.8)	20 (19.2)		102 (40.6)
Yes	65 (44.2)	84 (80.8)		149 (59.4)
**Body perception**			**<0.001**	
Adequate	38 (25.8)	4 (3.9)		42 (16.7)
Inadequate	109 (74.2)	100 (96.1)		209 (83.3)
**Body dissatisfaction**			**<0.001**	
No	33 (22.4)	6 (5.8)		39 (15.5)
Yes	114 (77.6)	98 (94.2)		212 (84.5)
**Intuitive eating – Total score**^**b**^	3.79 ± 0.74	3.68 ± 0.65	0.230	
**Intuitive eating – UPE Subscale**^c^*	3.83 ± 1.00	3.67 ± 1.00	0.156	
**Intuitive eating – EPR Subscale**^c^*	3.50 ± 1.37	3.13 ± 1.50	**<0.001**	
**Intuitive eating – RHSC Subscale**^c^*	3.50 ± 1.33	3.00 ± 1.17	**<0.001**	
**Intuitive eating – B-FCC Subscale**^c^*	3.67 ± 1.00	3.50 ± 1.00	**<0.001**	

Chi-square test. ^a^Fisher’s exact test. ^b^Student *t*-test. ^c^Mann-Whitney test. *Data expressed as p50 ± interquartile range. In bold: statistically significant difference (*p* < 0.05). *N* = 251. ^1^*N* = 244. ^2^*N* = 235. B-FCC: Body-food Choice Congruence. EPR: Eating for Physical Rather than Emotional Reasons. RHSC: Reliance on Hunger and Satiety Cues. UPE: Unconditional Permission To Eat.

In both the first and third years of the pandemic, overweight and obesity were more common in females (63.8% and 64.6%, respectively). Furthermore, the data also showed that, in both periods, both inadequate body perception and body dissatisfaction were present in a greater proportion in overweight and obese participants.

In the multiple analysis by binary logistic regression (Table 5), when the associations between the variables in the first year of the COVID-19 pandemic were evaluated, being female reduced the chances of being overweight or obese by 73.5% compared to males (OR = 0.265; CI = 0.113–0.623; *p* = 0.002), as well as tobacco use (91.8% reduction). Living in a marital relationship increased the chances of being overweight or obese by 21.2 times (OR = 21.229; CI = 4.663–96.645; *p* = <0.001), and this increase was also present in individuals who were concerned about weight gain, with 6 times more odds (OR = 6.202; CI = 2.645–14.593; *p* = <0.001), and body dissatisfaction, with 5 times more odds (OR = 5.368; CI = 1.045–27.574; *p* = 0.044).

**Table 5:** Multiple analysis according to nutritional status of undergraduate students at a university in southeastern Brazil in the first and third year of the COVID-19 pandemic, Vitória, 2020–2022.

Variables	Crude		Adjusted	
	*p*-value	OR (CI95%)	*p*-value	OR (CI95%)
**First year of the COVID-19 pandemic**				
**Sex**				
Male		1		1
Female	**0.018**	**0.506 (0.288–0.890)**	**0.002**	**0.265 (0.113–0.623)**
**Marital status**				
Do not live maritally		1		1
Live maritally	**<0.001**	**10.423 (3.818–28.453)**	**<0.001**	**21.229 (4.663–96.645)**
**Graduation area**				
Health		1		1
No health	**0.018**	**1.947 (1.122–3.380)**	0.238	1.522 (0.758–3.054)
**Practice of restrictive diets**				
No		1		1
Yes	**<0.001**	**2.742 (1.612–4.665)**	0.095	1.854 (0.899–3.821)
**Tobacco use**				
No		1		1
Yes	0.064	0.143 (0.018–1.124)	**0.047**	**0.082 (0.007–0.969)**
**Started cooking more**				
No		1		1
Yes	**0.009**	**0.458 (0.255–0.820)**	0.431	0.739 (0.348–1.570)
**Concern about weight gain**				
No		1		1
Yes	**<0.001**	**5.937 (3.105–11.353)**	**<0.001**	**6.202 (2.645–14.543)**
**Body perception**				
Adequate		1		1
Inadequate	**0.014**	**3.183 (1.266–8.008)**	0.074	3.002 (0.899–10.021)
**Body dissatisfaction**				
No		1		1
Yes	**0.002**	**6.584 (1.943–22.313)**	**0.044**	**5.368 (1.045–27.574)**
**Intuitive eating – total score**	**0.015**	**0.556 (0.348–0.891)**	0.634	0.844 (0.419–1.700)
**Intuitive eating – UPE Subscale**	**0.016**	**0.614 (0.414–0.913)**	0.540	1.197 (0.673–2.128)
**Third year of the covid-19 pandemic**				
**Sex**				
Male		1		1
Female	**0.017**	**0.504 (0.288–0.883)**	**<0.001**	**0.238 (0.109–0.521)**
**Marital status**				
Do not live maritally		1		1
Live maritally	**<0.001**	**6.673 (2.609–17.067)**	**<0.001**	**9.748 (3.045–31.207)**
**Practice of restrictive diets**				
No		1		1
Yes	**<0.001**	**3.221 (1.728–6.004)**	**0.008**	**2.887 (1.312–6.357)**
**Alcohol use**				
No		1		1
Yes	**0.016**	**1.870 (1.124–3.108)**	0.123	1.675 (0.870–3.226)
**Concern about weight gain**				
No		1		1
Yes	**<0.001**	**5.298 (2.948–9.523)**	**<0.001**	**4.081 (1.920–8.677)**
**Body perception**				
Adequate		1		1
Inadequate	**<0.001**	**8.716 (3.003–25.293)**	**0.023**	**3.984 (1.207–13.153)**
**Body dissatisfaction**				
No		1		1
Yes	**0.001**	**4.728 (1.902–11.755)**	0.051	2.866 (0.995–8.257)
**Intuitive eating – EPR subscale**	**0.001**	**0.638 (0.485–0.841)**	0.802	0.948 (0.627–1.435)
**Intuitive eating – RHSC subscale**	**<0.001**	**0.583 (0.447–0.759)**	0.092	0.729 (0.505–1.053)
**Intuitive eating – B-FCC subscale**	**<0.001**	**0.529 (0.381–0.732)**	**0.015**	**0.567 (0.360–0.894)**

Binary logistic regression crude and adjusted. Hosmer e Lemeshow test: First year—0.416; Third year—0.481. R square nagelkerke: first year—0.454; third year—0.475. In bold: statistically significant difference (*p* < 0.05). B-FCC: body-food choice congruence. EPR: eating for physical rather than emotional reasons. RHSC: reliance on hunger and satiety cues. UPE: unconditional permission to eat. OR: odds ratio. CI: confidence interval.

In the analysis of the associations between the variables obtained in the third year of the pandemic, the practice of restrictive diets (OR = 2.887; CI = 1.312–6.357; *p* = 0.008) and inadequate body perception (OR = 3.984; CI = 1.207–13.153; *p* = 0.023) more than doubled the participants’ chances of being overweight or obese. Concern about weight gain (OR = 4.081; CI = 1.92–8.677; *p* = <0.001) and living in a marital relationship (OR = 9.748; CI = 3.045–31.207; *p* = <0.001) increased the chances of being overweight or obese by 4 and 9 times, respectively. On the other hand, being female reduced these odds by 76.2% (OR = 0.238; CI = 0.109–0.521; *p* = <0.001), and congruence in body-food choices (B-FCC subscale of intuitive eating) by 43.3% (OR = 0.567; CI = 0.36–0.894; *p* = 0.015).

## 4 Discussion

The results of this study showed that being female and using tobacco reduced the chances of university students being overweight/obesity in the first year of the COVID-19 pandemic. Living in a marital relationship, worrying about weight gain and being dissatisfied with body image significantly increased these odds in the same period. In the third year of the pandemic, the practice of restrictive diets, inadequate body perception, concern about weight gain and living in a marital relationship increased the odds of overweight/obesity in this population. Being female and eating more intuitively, with congruence in body-food choices, contributed to reducing these odds. These results are relevant, since the changes that occurred during the confinement period had an impact on health behaviors, resulting in weight gain (24–26).

When we analyzed our non-probabilistic sample of students, we found that it was representative of the total population, due to its similarity to the sociodemographic profile described in the 5th National Survey on the Socioeconomic and Cultural Profile of Undergraduates at Federal Higher Education Institutions (IFES), carried out by the National Association of Directors of Federal Higher Education Institutions (ANDIFES) (27). The sample was predominantly female, similar to the survey published by ANDIFES. The representative participation of the female public was also observed in higher percentages than the survey in other studies carried out with university students during the COVID-19 pandemic, such as the work by Liboredo et al. (28) carried out in Minas Gerais, by Santana et al. (29) in the Northeast, and in a study carried out in Rio Grande do Sul (30). There was also a predominance of individuals in our sample who were not living in a marital relationship, similar to the data from the mentioned census.

This study found that being female reduced the chances of being overweight during the pandemic. Although women are usually more prone to weight gain (31), our study showed that being female had a protective effect against excess weight, both in the first and third year of the pandemic, which has also been observed in other studies. The study by Barros et al. (32) found a higher prevalence of overweight among men compared to women, as did the study by Sousa and Barbosa (33). In surveys of university students in Bahia, the authors showed an increase in the frequency of overweight in men over a 4-year period (2010–2014); in relation to women, there was no significant difference (34–36).

Tobacco use also significantly reduced the chances of overweight and obesity, demonstrating a relationship between these two variables, which has already been shown in other studies (37–39). The study by Tamborindeguy and Moraes showed that individuals who stopped smoking not only gained weight but also changed their nutritional status, which may be associated with changes in taste, anxiety and changes in eating behavior. Although in our study smoking reduced the chances of overweight/obesity, cigarette use is a risk factor for morbidity and mortality and is not considered a healthy practice for managing excess weight (40).

The data also showed that living in a marriage significantly increased the chances of overweight/obesity in the first year of the pandemic. It is possible that individuals who are not married are more careful about their body weight in order to attract partners. Also, after marriage, individuals may have increased family responsibilities resulting in less time for physical exercise. It is also more likely that there will be a change in eating patterns and that constancy in weight control will decrease (41). All these issues taken together may have contributed to the association between living as a couple and greater chances of being overweight.

Worrying about weight gain and being dissatisfied with their body image were also factors that increased the chances of university students being overweight during the first survey. Nutritional status and the practice of weight loss diets were the variables associated with body image in the study by Costa and Vasconcelos (42). Similarly, Bosi et al. (43) found an association between body dissatisfaction and BMI, where 82.9% of university students who had a moderate/severe score on the Body Shape Questionnaire, used to assess body image concerns, were eutrophic.

The data from the second moment in our study was collected at the end of the social distancing measures and the return to face-to-face activities at the universities. This period had to be chosen so that the data collected could reflect the changes that occurred during the pandemic, as well as making it possible to verify the predictors of risk and protection of nutritional status during this scenario. In this second collection, practicing restrictive diets, as well as having inadequate body perception, worrying about weight gain and living in a marital relationship increased the chances of overweight/obesity in this population. Concern about weight gain can lead to dysfunctional eating behaviors, such as eating to cope with negative feelings, which reduce the intrinsic perception of hunger and satiety and lead to increased food consumption (44). Similarly, the practice of restrictive diets results in inappropriate eating behaviors and is associated with a troubled relationship between food and the body (45).

Di Renzo et al. (46) reported in their study that isolation, lack of stimuli, boredom and a change in eating habits affected 86% of respondents who reported not being able to control their eating sufficiently during the pandemic. In addition, almost half of those interviewed said they felt anxious because of their eating habits. Sisto et al. (47) also showed a relationship between symptoms of anxiety and depression and snack consumption, as well as increased hunger and impulsive eating, as a result of the negative impact of suffering on eating. The state of depression or the worsening of this condition are precursors to weight gain (48). Daniel and collaborators (49) revealed in their study that an increase in uncontrolled eating behavior was a predictor of weight gain during the pandemic. Thus, these behaviors are relevant influences on food choices and, consequently, on BMI (50).

Also, in the second collection period, being a woman and eating more intuitively contributed to a reduction in the chances of being overweight. Intuitive eating is an adaptive style of eating that involves a strong connection with physiological signals of hunger and satiety, so that the individual eats in response to these signals and not due to external influences, as occurs mainly in restrictive diets (51). It is related to weight stability (12) and negatively related to higher weight status (18, 52). Camilleri and Méjean (52) showed a strong inverse association between intuitive eating and overweight and obesity. These associations were also observed between the three subscales—EPR, RHSC and B-FCC. In fact, obese individuals have less confidence in hunger and satiety signals, and eat less for physical reasons and more for emotional reasons, which explains why emotional eating is associated with increased body weight (53).

An interesting finding in our study was the high proportion of body dissatisfaction among underweight/eutrophic individuals. This result was also found by Da Costa et al. (54), in which most of the sample of young adults were eutrophic, but dissatisfied with their bodies, with over 70% of them wanting a smaller figure. The results found in the study by Miranda et al. (55), with university students in Juiz de Fora, showed that the prevalence of body image dissatisfaction was high, reaching 76.6%. A similar finding was found in the study by Coqueiro et al. (56), with a prevalence of 78.8%.

In his study, Laus (57) describes the ability of the mass media to produce changes in body image and dietary choices in young university students, through exposure to idealized images. The use of social networks is widespread among university students (58), and the social distancing measures caused by the COVID-19 pandemic may also have been a contributing factor, since face-to-face meetings have been replaced by virtual ones, which contributes to an increase in body dissatisfaction even in individuals weighing within normal parameters (59).

When comparing data from the first and third year of the pandemic, we found that students reported weight gain throughout this period. Other studies have also shown a similar picture during the pandemic. In the Italian population, weight gain was observed in 48.6% of individuals (60), in Poland in 30% of the population studied (61) and in 22% of participants in the study by Zachary et al. (62). The reasons for these changes ranged from an increase in portion size and the number of meals consumed to a decrease in physical activity (63). Lima et al. (64) observed weight changes in 74.4% of university students, and inadequate BMI in 21.8% of them during the period of social distancing. The prevalence of a sedentary lifestyle was 37.8%. As for dietary changes, 44.2% of students reported an increase in portioning, 47% in the volume of meals, and an increase in the consumption of fast food and ultra-processed foods. In Turkey, there was a 35% increase in the rate of weight gain over a period of 10 weeks after the first blockade practice (65).

There was also an increase in intuitive eating between the two periods, both in the total score and in the UPE and B-FCC subscales. On the other hand, the proportion of university students dissatisfied with their body image decreased significantly, which may be associated with this increase in the intuitive eating score. Van Dyke and Drinkwater (18) showed in their study that higher levels of intuitive eating are associated with lower levels of concerns about body shape and weight, regardless of BMI. Since the main purpose of intuitive eating is a good relationship with food and the body, it is inferred that the practice is capable of reducing the chances of dieting and increasing body satisfaction (66). The UPE subscale reflects the permission to eat when hungry, without the need to follow extrinsic food rules and/or exclude forbidden foods. It is understandable that individuals with higher scores on this subscale allow themselves to enjoy a greater variety of foods, without judgment and guilt (12).

In addition, in both the first and second surveys, we observed lower intuitive eating scores in overweight individuals. Other studies have shown similar results, where both the total intuitive eating score and its subscales were lower in overweight and obese individuals, indicating less intuitive eating in this group (52, 67). These studies corroborate the association observed between more intuitive eating and lower chances of being overweight in our study.

Penaforte et al. (1) showed that students with higher levels of perceived stress had higher scores for emotional eating and uncontrolled eating habits. Thus, it is clear that in stressful situations, such as the pandemic, emotional factors determine food choices. Caferoglu and Toklu (68) revealed that an increase in intuitive eating patterns was related to a reduction in BMI, so that a higher intuitive eating score and also higher scores on the subscales were related to a lower BMI, which corroborates the main findings of our study.

It is important to highlight, however, that although intuitive eating is associated with a lower BMI (52, 53), this way of eating is not capable of, in isolation, preventing weight gain, since this gain is multifactorial, and potentially greater in an adverse context, such as the pandemic (24–26). Thus, more intuitive eating may favor greater weight stability (12), and may, therefore, have helped to reduce the impact of the pandemic on weight gain. In this way, people who ate more intuitively, guided by internal rather than external signs of hunger and satiety, partially resisted the context that favored weight gain (stress, changes in diet and physical inactivity).

This study provides important information that can help to mitigate the damage to health caused by the pandemic, as well as to develop strategies to prevent excessive weight gain in potentially stressful scenarios. Also noteworthy is the repeated measures observational study, which made it possible to monitor the variables analyzed over two years and thus assess the impact of social restrictions on students’ nutritional status. However, the study has some limitations. Data was collected using an online questionnaire, and weight and height measurements were self-reported. However, several epidemiological studies used a similar methodology in the same period, without this significantly damaging the research’s inferences. In addition, data collection using virtual means simplifies fieldwork, as well as being low cost (69) and enabling greater participation in the research.

## Data availability statement

The original contributions presented in the study are included in the article/supplementary material, further inquiries can be directed to the corresponding author.

## Ethics statement

The studies involving humans were approved by Research Ethics Committee of the Federal University of Espirito Santo (UFES). The studies were conducted in accordance with the local legislation and institutional requirements. The participants provided their written informed consent to participate in this study.

## Author contributions

CS: Writing – original draft. LS: Writing – review & editing. GS: Writing – original draft. MC: Writing – review & editing. YS: Writing – original draft. FS: Writing – review & editing.
